# Toward panoramic *in situ* mapping of action potential propagation in transgenic hearts to investigate initiation and therapeutic control of arrhythmias

**DOI:** 10.3389/fphys.2014.00337

**Published:** 2014-09-08

**Authors:** Miroslav Dura, Johannes Schröder-Schetelig, Stefan Luther, Stephan E. Lehnart

**Affiliations:** ^1^Heart Research Center GöttingenGöttingen, Germany; ^2^Department of Cardiology and Pulmonology, University Medical Center GöttingenGöttingen, Germany; ^3^Biomedical Physics, Max Planck Institute for Dynamics and Self-OrganizationGöttingen, Germany; ^4^Institute for Nonlinear Dynamics, Georg-August-Universität GöttingenGöttingen, Germany; ^5^German Centre for Cardiovascular Research (DZHK), partner site Göttingen (DZHK-GOE)Göttingen, Germany

**Keywords:** ventricle, heart, optical mapping, voltage imaging, panoramic imaging

## Abstract

To investigate the dynamics and propensity for arrhythmias in intact transgenic hearts comprehensively, optical strategies for panoramic fluorescence imaging of action potential (AP) propagation are essential. In particular, mechanism-oriented molecular studies usually depend on transgenic mouse hearts of only a few millimeters in size. Furthermore, the temporal scales of the mouse heart remain a challenge for panoramic fluorescence imaging with heart rates ranging from 200 min^−1^ (e.g., depressed sinus node function) to over 1200 min^−1^ during fast arrhythmias. To meet these challenging demands, we and others developed physiologically relevant mouse models and characterized their hearts with planar AP mapping. Here, we summarize the progress toward panoramic fluorescence imaging and its prospects for the mouse heart. In general, several high-resolution cameras are synchronized and geometrically arranged for panoramic voltage mapping and the surface and blood vessel anatomy documented through image segmentation and heart surface reconstruction. We expect that panoramic voltage imaging will lead to novel insights about molecular arrhythmia mechanisms through quantitative strategies and organ-representative analysis of intact mouse hearts.

## Introduction: molecular principles of AP propagation derived from transgenic hearts

**Figure 1:**
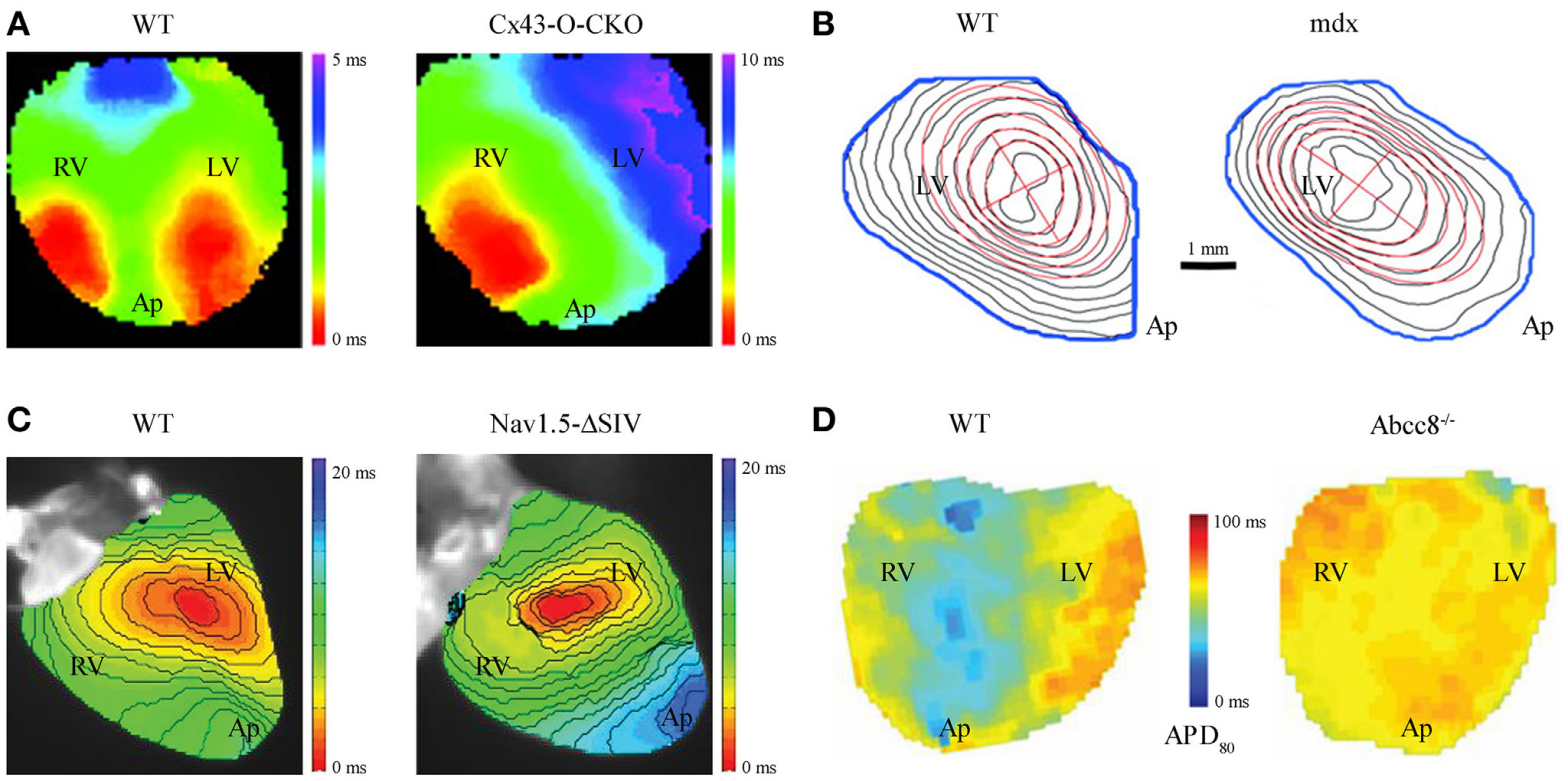
**Altered impulse propagation characterized by VSDI in transgenic mouse hearts**. **(A)** Epicardial activation during spontaneous sinus rhythm in wild-type (WT) vs. cardiac specific Cx43 knockout mouse hearts, the latter a model of gradual loss (Cx43-O-CKO). Whereas WT hearts typically show 2 distinct sites of AP activation (“breakthroughs”) each for the left (LV) and right (RV) ventricle as seen on the anterior heart surface, Cx43-O-CKO mouse hearts show aberrant activation patterns (e.g., only one epicardial breakthrough or multiple fragmented sites of AP activation) leading to overall reduced transversal CV (WT 0.40 ± 0.02 vs. Cx-O-CKO 0.21 ± 0.02 m/s; Figure reproduced from Morley et al., 2005, Copyright 2005 National Academy of Sciences, U.S.A.). **(B)** Isochrone maps at 0.5 ms resolution in WT vs. dystrophin-deficient mdx hearts show electrical activation delay during LV pacing at 100 ms cycle length (red cross indicates epicardial pacing site at the apparent LV center). CV in mdx hearts is significantly reduced, particularly in the transversal direction relative to the main fiber orientation (WT 0.48 ± 0.03 vs. mdx 0.35 ± 0.07 m/s), and apparent through denser isochrone fits (red ellipses) due to selective loss of Nav1.5 channels at the lateral surface membrane in myocytes (Figure reproduced from Petitprez et al., 2011 with permission of Lippincott Williams & Wilkins, Inc.). **(C)** LV activation during pacing at 120 ms cycle length in Nav1.5-ΔSIV knock-in hearts expressing a C-terminally truncated channel peptide. Nav1.5-ΔSIV hearts display an LV conduction defect caused by select loss of Nav1.5 targeting to the lateral surface membrane of LV myocytes resulted in decreased longitudinal CV (WT 0.70 ± 0.04 vs. Nav1.5-ΔSIV 0.54 ± 0.03 m/s; Figure reproduced from Shy et al., 2014 with permission of Lippincott Williams & Wilkins, Inc.). **(D)** Action potential duration (APD) maps during ventricular pacing and adrenergic stimulation with isoprotenerol and the selective phosphodiesterase type 4 inhibitor rolipram (each 10 μM) in WT vs. Abcc8^−/−^ knockout hearts, i.e., deficient for the K_ATP_ channel containing pancreatic sulfonylurea receptor subunit (SUR1) devoid of SUR1 containing K_ATP_ channels. The absence of SUR1 protein in Abcc8^−/−^ hearts disrupts physiological APD shortening as occurs in WT hearts during sustained adrenergic stimulation identifying an essential role of SUR1-containing K_ATP_ channels for ventricular action potential regulation (Figure reproduced from Arakel et al., 2014 with permission of Company of Biologists Ltd.). Please note different cardiac orientations in **(A–D)**; LV, left ventricle; RV, right ventricle; Ap, apex.

Molecular physiology and genetic engineering studies have established complex genetic and cellular determinants of impulse propagation in mouse models. Four distinct molecular principles were identified: connexin channels at gap junctions control solute flux between myocytes and electrical current flow throughout the myocardial syncytium (Figure 1A); cardiac Na^+^ channels (Nav1.5) expressed at high density in myocyte membranes determine voltage-dependent activation of AP generation and propagation; and intracellular ryanodine receptors (RyR2s) activated through voltage-dependent Ca^2+^ channels (Cav1.2s) shape the intracellular Ca^2+^ transient during excitation-contraction (EC) coupling. In addition, AP duration (APD) and the stability of cardiac repolarization are regulated by distinct K^+^ channel types, e.g., during ischemia activation of ATP-sensitive K^+^ channels (K_ATP_) leads to APD shortening thereby preventing potentially detrimental intracellular Ca^2+^ overload. While each principle contributes to impulse generation, propagation and/or modulation in heart tissue, the molecular mechanisms of AP modulation and disease changes are exceedingly complex and depend on thorough analysis in intact heart tissue based on non-invasive imaging methods.

To investigate intact hearts, voltage-sensitive dye imaging (VSDI) or multiparametric imaging (e.g., VSDI combined with a Ca^2+^ dye) have become a central strategy to study impulse propagation in transgenic mouse hearts in which EC coupling cycles occur an order of magnitude faster than human heart. High VSDI based resolution has allowed the functional characterization of anisotropic impulse propagation in mouse hearts. This has revealed the physiological relevance of select Nav1.5 channel membrane targeting mechanisms and led to the concept of distinct, locally confined pools of Nav1.5 channels at the intercalated disc (ICD) vs. the lateral cell membrane (Petitprez et al., 2011). Notably, the consequences of Nav1.5 patient mutations are currently unclear at the tissue level. However, the mdx mouse model of muscular dystrophy, featuring a loss of dystrophin, shows a select loss of Nav1.5 only in the lateral myocyte membrane and is associated with a significantly decreased conduction velocity (CV) transversal to the main myocyte fiber orientation (Petitprez et al., 2011; Figure 1B). This led to the realization of differential targeting of the Nav1.5 protein complex, which binds to dystrophin only at the lateral cell membrane. Furthermore, deletion of the putative C-terminal PDZ-binding targeting domain that localizes Nav1.5 channel complexes at the lateral myocyte membrane led to significant reduction of transversal CV (Shy et al., 2014; Figure 1C). Taken together, these findings strongly support the notion that the lateral myocyte membrane and the ICD exert significantly different roles during cardiac impulse propagation, while each is necessary for normal anisotropic conduction.

In the ICD membrane portion Nav1.5 channels associate with gap junction connexin hemichannels. Hence, pathological remodeling of connexin channels may contribute to Nav1.5 dysfunction (Sato et al., 2011; Delmar, 2012). Changes in connexin expression and function are generally associated with an increased propensity for arrhythmias. In particular, loss of the main ventricular gap junction protein connexin43 (Cx43) significantly decreases CV and disrupts the physiological activation pattern in adult mouse hearts (Gutstein et al., 2001; Morley et al., 2005; Figure 1A). Moreover, changes in Cx43 expression were associated with remodeling of major ionic currents contributing to cardiac repolarization (Danik et al., 2008) and a significant reduction in Nav1.5 current amplitude (Jansen et al., 2012), predicted to increase electrical tissue heterogeneity and the propensity for arrhythmias. Consistent with the latter observations, the human Cx43-I130T mutation was shown to decrease CV and cause arrhythmias in a mouse model (Kalcheva et al., 2007). Finally, abnormal impulse generation and propagation may occur during diastolic Ca^2+^ overload from leaky sarcoplasmic reticulum Ca^2+^ stores caused by RyR2 missense mutations as demonstrated in knockin mice, which reproduce the characteristic stress-induced arrhythmias observed in patients (Cerrone et al., 2007; Lehnart et al., 2008).

To address emergent genetic models and corresponding imaging strategies for transgenic hearts, we focus on the important juncture of VSDI for the study of molecular disease mechanisms through state-of-the-art strategies in the adult mouse heart. While VSDI approaches for panoramic imaging have been realized, impulse propagation was exclusively characterized in larger mammalian hearts, in particular rabbit (Li et al., 2009; Ripplinger et al., 2009) and pig (Chattipakorn et al., 2001; Kay and Rogers, 2006; Kay et al., 2006; Rogers et al., 2007; Bourgeois et al., 2012). As cardiac electrophysiology in general and clinical translation in particular depend critically on 3D tissue behaviors, including quantification of conduction changes and spatial mechanisms of arrhythmia onset, here we consider the potential of panoramic imaging of transgenic mouse hearts to elucidate physiological disease mechanisms. Finally, the prospect to apply panoramic imaging approaches to new concepts with important therapeutic implications will be discussed: 3D multi-functional integumentary membranes (3D-MIMs) and strategies for low-energy anti-arrhythmia pacing (LEAP).

## Optical action potential measurements in mouse hearts

Complex mechanisms of AP generation, propagation, and modulation underlie anisotropic impulse spread, i.e., the physiological form of mammalian heart tissue excitation (Figure 1A; WT). These mechanisms reflect the specific 3D organization and orientation of all excitable cells including cardiac myocytes and Purkinje fibers at select tissue regions during each cardiac cycle. Furthermore, pathological changes may affect cell and/or tissue functions and thereby lead to altered AP spread and increased arrhythmia propensity (Figures 1B,D). Hence, quantitative methods to characterize cardiac AP spread in space and time, and at the scales of small mouse hearts are needed. In addition, transgenic mouse models are critical to establish new molecular concepts about fundamental arrhythmia mechanisms or to explain increased arrhythmia propensity, particularly for genetic patient syndromes. For example, genetic loss-of-function models are highly valuable to understand the specific molecular roles of connexin or dystrophin, and each for specific cells and tissues during cardiac impulse propagation (Figures 1A,B). Notably, while the shape and duration of the mouse AP differs significantly from larger mammalian hearts, the rate adaptation of APD and restitution properties show surprising similarities (Knollmann et al., 2001, 2007). Taken together, optical AP measurement and additional electrode-based methods (see below) can be used and combined to characterize transgenic mouse hearts.

### Advantages and disadvantages of different methods for AP measurement in cardiac tissue

Glass or metal microelectrodes were initially used to measure APs (Omichi et al., 2000). Nowadays monophasic action potential (MAP) electrodes are used in mouse heart (Knollmann et al., 2001; Chiello Tracy et al., 2003). MAP recording is an important reference technique, but is limited by spatial resolution and electrode flexibility particularly if applied to rapidly contracting mouse hearts (Kadish, 2004; Kondo et al., 2004). To enable spatial analysis of tissue activation, microelectrode arrays were developed (Reppel et al., 2004; Meyer et al., 2010). Typical microelectrode arrays consist of 32–252 electrodes positioned in a geometric grid and allow multi-site recording from mouse hearts at the cost of regional tissue-grid interactions (Lehnart et al., 2006; Zhang et al., 2013). In contrast to electrode-based point measurements, voltage-sensitive fluorescent dye imaging (VSDI) enables continuous, non-invasive optical mapping of cardiac AP propagation throughout tissue regions from the heart surface. Potentiometric, voltage-sensitive dyes intercalate directly into the cell membrane and report changes of the transmembrane potential as spectral shift in light emission. Potential limitations of VSDI, baseline drift of the resting potential and motion artifacts interfering with AP repolarization can be overcome by ratiometric recording through two emission wavelengths (Knisley et al., 2000). In general, a sufficiently high signal to noise ratio (SNR) has to be established, e.g., di-4-ANEPPS is typically used in mouse hearts to record fractional changes at 10%/100 mV or higher. VSDI is limited by the spatial resolution given by the sensor size of the detector, inadvertent dye phototoxicity, and potential movement artifacts, which can be controlled by uncoupling agents (BDM or blebbistatin; Fedorov et al., 2007; Lou et al., 2012; Swift et al., 2012) and/or ratiometric imaging. An important advantage is that transmembrane voltage changes are directly recorded within millisecond or higher temporal resolution.

### Analysis of optical mapping data from mouse hearts

Due to the high resolution of optical voltage data, detailed information about depolarization and repolarization behaviors of intact heart tissue can be extracted. In mouse heart, millisecond or faster temporal resolution of modern CMOS cameras (100 × 100 pixels; 1–10 kHz frame rate) and a planar resolution of 50–100 μm are achieved. Each sensor pixel records the fluorescent signal average of multiple cells. The tissue-specific propagation of epicardial AP wave fronts can be analyzed during sinus rhythm (Figure 1A) or during external pacing (Figure 1B). Isochrone time maps reveal important spatiotemporal information about dynamic AP spread including the origin(s), direction, and velocity of cardiac tissue activation. For quantitation of tissue activation, the half-maximal signal intensity and/or the point of fastest upstroke velocity can be used. Impulse propagation can be further characterized through vector maps, where each vector represents the local conduction velocity (CV) and propagation direction. However, fast activation spread in smaller tissue regions may last only 1–2 ms. In this case, plane fitting of regional pixel areas (e.g., 7 × 7 pixels) and activation times can be used, and CV amplitude and direction determined as average vector for a particular region (Laughner et al., 2012). In addition, the minimum and maximum CV relative to the main longitudinal and transversal myocyte fiber orientations can be analyzed through CV angle binning or ellipse fitting algorithms (see Figure 1B; Petitprez et al., 2011). Taken together, quantitative analysis through CV mapping can lead to important insights about local activation patterns and/or regions of conduction slowing or block.

Furthermore, both physiological and pathological changes of cardiac repolarization can be analyzed. Abnormal AP formation (e.g., after depolarizations) as mechanism of triggered arrhythmias, abnormal AP propagation (e.g., CV heterogeneity between adjacent regions), and reentry contribute to arrhythmia initiation and/or propagation. Optical signals recorded by individual pixels can be analyzed to determine local APD, e.g., repolarization at 80% of the signal amplitude (Figure 1D). Similar to CV analysis, vector maps of APD gradients can be generated to investigate dynamic AP spread and tissue heterogeneities. Reentry leading to spiral waves facilitated by beat-to-beat variability and/or the slope of electrical restitution can be recorded and analyzed as needed, e.g., to estimate the propensity for arrhythmias. Taken together, AP repolarization parameters and restitution slope can be derived from VSDI data and repolarization changes associated with tissue-specific arrhythmia mechanisms (Wilson and Rosenbaum, 2007).

## Panoramic heart imaging and surface reconstruction analysis

**Figure 2:**
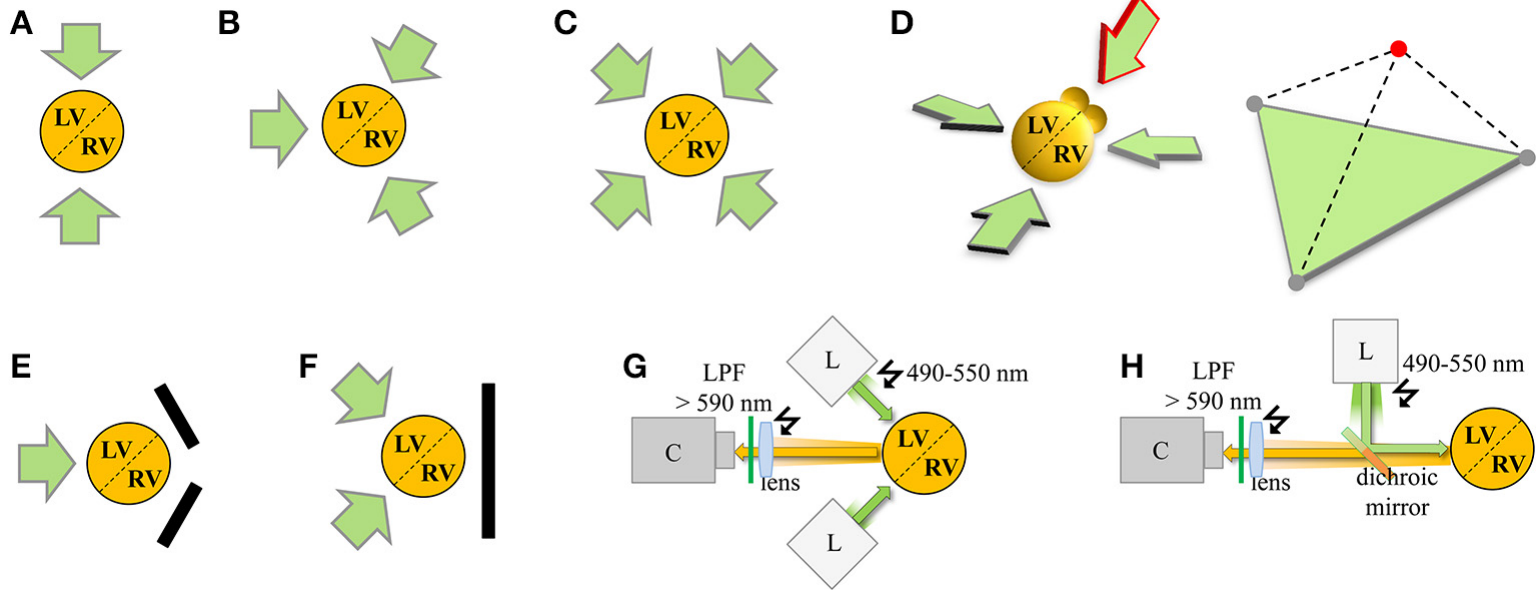
**Schematic summary of VSDI configurations for panoramic cardiac imaging**. The orange circle represents the total emitted red-shifted fluorescence voltage signal emanating from the cardiac 3D object comprised of the left (LV) and right (RV) ventricles, and anatomically delimited by epicardial/septal blood vessels (dashed line). Each green arrow represents an individual VSDI camera configuration. Specific configurations of panoramic multi-camera setups assuming a central heart axis of interest are indicated as shown (for associated excitation light configurations please refer to **G,H**). **(A)** 2-camera configuration for pig heart recording according to Chattipakorn et al. (2001); **(B)** 3-camera configuration for rabbit hearts according to Qu et al. (2007), Evertson et al. (2008); **(C)** 4-camera configuration for pig hearts according to Kay and Rogers (2006). **(D)** Theoretical 3D extension based on the camera configuration in **(B)** with 3 in-plane cameras imaging the ventricles and 1 out-of-plane camera imaging the atria (red arrow). The 3D camera configuration is summarized in the right tetrahedron by 3 gray circles in the green base plane and the red circle on top for simultaneous imaging of the atria. In addition, variants replacing cameras with mirrors were developed as **(E)** 1-camera/2-mirror configuration by Lin and Wikswo (1999), Bray et al. (2000) and **(F)** 2-camera/1-mirror configuration by Kay et al. (2004). **(G,H)** Each VSDI camera requires at least 1 associated light source (e.g., Xenon or Mercury arc lamp, LEDs, or halogen lamp; sometimes applied through multiple optical fibers). Adjustment of the excitation light beam ‘L’ relative to the 3D imaging object and optical collection of the emitted light signal on the camera sensor ‘C’ occurs through a lens and appropriate filters, optimized either for separate light paths **(G)** or combining light paths through a dichroic mirror **(H)**. Green arrow, excitation light beam; orange arrow, emitted light signal from red-shifted dye like di-4-ANEPPS; LPF, long pass filter. Note that an additional conventional camera as described in the main text is typically used for anatomical surface texture recording and geometrical surface reconstruction (not shown for clarity).

An important limitation of single camera imaging setups is that the AP propagation (and additional multiparametric readouts like Ca^2+^ transients) cannot be analyzed continuously throughout the whole surface of the mouse heart, especially the spatially complex 3D impulse spread during fast arrhythmias. In addition, the high curvature-to-volume ratio of mouse hearts distorts fluorescent signals originating from shallow angles of peripheral boundaries, limiting the effective ROI of single camera approaches (e.g., Figure 1B). To overcome these limitations, panoramic mouse heart imaging is currently being developed by several groups based on previous application in larger species (Figures 2A–C). Early panoramic measurements combined one CCD camera (128 × 64 pixels; 335 fps) with 2 planar mirrors to gain optical representation of the entire heart surface (Figure 2E), but the resulting fluorescent images recorded from different heart regions didn't allow for continuous tracking of impulse spread. This was overcome through reconstruction of the heart surface geometry and surface (epicardial) correlation of fluorescent signals (Bray et al., 2000). Panoramic imaging was further improved by simultaneous use of several detectors, thus replacing some (Kay et al., 2004; Figure 2F) or all mirrors with cameras (Figures 2A–C, e.g., Qu et al., 2007; Rogers et al., 2007). Later on geometrical reconstruction of the heart surface was further facilitated through a separate high resolution camera for epicardial image acquisition. Together with sensor calibration this allowed analysis of VSDI data related to the geometry of the heart surface (e.g., CV; Kay et al., 2004; Qu et al., 2007; Lou et al., 2008). Geometrically registering fluorescent voltage signals with the surface anatomy of the heart (texture mapping) enables spatially continuous visualization of the electrical activation in a tissue-representative manner.

Modern panoramic imaging setups position 3 photo-diode arrays (PDAs; Figure 2B; Lou et al., 2012) or 3–4 CCD cameras (Figure 2C; Kay et al., 2006; Evertson et al., 2008; Bourgeois et al., 2012) symmetrically around the heart. A conventional CCD camera documents the heart surface at 5–10° steps for anatomical geometry reconstruction. For example, panoramic imaging showed defibrillation mechanisms in pig hearts (Chattipakorn et al., 2001), short-lived rotors as a mechanism of VF (Kay et al., 2006), right ventricular sites of wave break during transition to VF (Bourgeois et al., 2012), and interactions of reentry rotors with the border zone in post-MI rabbit hearts (Li et al., 2009; Ripplinger et al., 2009). However, comprehensive organ-representative investigation of molecular arrhythmia mechanisms in the genetically tractable mouse heart has remained challenging. For instance, with modern PDA detectors (16 × 16 elements) and optics suitable for adult mouse heart imaging (6 × 6 mm) the spatial resolution is approximately 0.4 mm (Rosenbaum, 2002). We and others (Leaf et al., 2008; Petitprez et al., 2011; Remo et al., 2011; Arakel et al., 2014; Shy et al., 2014) have used fast cameras with millisecond or higher resolution to capture activation patterns in different mouse models (Figures 1A–D). The CMOS sensors acquire signals with high SNR and improved dynamic range (comparable to PDAs) with up to 10 kHz sampling frequency and a spatial resolution similar to CCD cameras (100 × 100 pixels). This makes CMOS cameras an obvious candidate for VSDI setups and multiparametric readouts for arrhythmia analysis in mouse heart, e.g., to dissect the molecular mechanisms of complex phenotypes like the long-QT syndrome. However, the use of CMOS cameras for panoramic imaging is currently limited by cost as 3 or more cameras are needed (Figures 2B–D).

## Challenges for panoramic imaging and emerging analysis concepts

Interpretation of panoramic imaging experiments depends critically on the quality of the raw fluorescence data sets. First, sufficient SNR must be established, which includes optimizing the optical configuration and light sources to achieve homogeneous tissue illumination and sufficient dye excitation in cardiac tissue (Figures 2G,H). While the signal can be improved by post-processing through a number of strategies (data masking, digital noise filtering, baseline drift corrections, normalization; Lou et al., 2008), these are usually not sufficient to remove original signal artifacts. For instance, non-linear drift of the signal baseline on a pixel-by-pixels basis is difficult to remove and can significantly distort any analysis of local APD and repolarization. Baseline drift is influenced by miscellaneous factors including unequal dye loading and changes in tissue thickness. Contraction uncoupling agents like blebbistatin may cause swelling of heart tissue (Kay et al., 2004) and small heart movements may contribute to imprecise anatomical reconstruction, considering that image acquisition and surface texture mapping are usually not synchronized.

Consequently, it is important to customize and optimize VSDI setups to systematically eliminate potential sources of signal artifacts. While several methods exist to correct in-plane tissue motion, e.g., through image registration (Rohde et al., 2005) or wavelet multiresolution analysis (Asfour et al., 2011), these methods are not generally sufficient to correct small local motion artifacts due to microscopic movements of the heart or its parts. Laughner et al. (2012) tracked cardiac surface movements with high spatiotemporal resolution using structured light illumination. Combined with VSDI this promises to digitally correct artifacts in fluorescent raw data from microscopic heart movements. Due to the high resolution this technique might be applied in real-time on a pixel-by-pixel basis. In summary, future data analysis strategies may allow for off-line or real-time motion correction in the absence of contraction uncoupling agents at high resolution, which may further improve panoramic mouse heart imaging.

## Extension to new pathophysiologically relevant 3D technologies

Current experimental and clinical electrophysiology is based on contact-point-catheters or contact-2D-sheet-electronics, which are not sufficient as chronic high resolution sensor interface for 3D epicardial or endocardial surface applications. Thus, to enable uniform multiplex contacts for precision measurements through chronic implants, new materials and stretchable electronic devices have been developed (Sekitani et al., 2008; Kim et al., 2011a; Kaltenbrunner et al., 2013). In addition, recent therapeutic device strategies exploit endogenous electrophysiological 3D tissue properties of the heart (Luther et al., 2011). Hence we extend our considerations to these important 3D therapeutic concepts: device integration with the cardiac surface through 3D-MIMs and low-energy anti-arrhythmic pacing (LEAP) for arrhythmia control.

### New sensor materials and systems for cardiac 3D surface integration

Since the cardiac electrical syncytium moves during each EC cycle, the ideal material/heart interface would non-invasively interact with the heart surface. Recently, flexible sheets were developed, which contain miniaturized electronic devices (Kim et al., 2012), and were further improved to build a multifunctional semiconductor system with defined 3D configurations of thin elastic membranes that precisely match the shape of the rabbit heart (Xu et al., 2014). These devices exploit ultrathin mechanical designs and active silicone electronic materials with extreme deformability and reversible elastic responses to large strain deformations at orders of magnitude smaller than the dimensions permitted by conventional technologies (Kim et al., 2011b). Proof-of-concept experiments established a flexible artificial membrane platform containing deformable semiconductor arrays of multiparametric sensors, electronic and optoelectronic components (Xu et al., 2014). Importantly, the transparent and miniaturized nature of the integumentary membrane system (3D-MIMs) allows for simultaneous optical mapping of 2D voltage activation, e.g., for methodological validation (Xu et al., 2014). Hence, the panoramic voltage mapping strategies discussed above may further aid the validation and upward translation of 3D-MIMs to be applied to the whole heart and in an *in vivo* context. The latter was in principle demonstrated in open-chest rodent models (Kim et al., 2011a, 2012). Furthermore, existing diagnostic concepts, i.e., catheter-based measurements or interventions and the new 3D-MIMs system can be conceptually integrated and further translated through panoramic heart imaging.

### New therapeutic anti-arrhythmic concepts (LEAP)

External and internal cardioverter defibrillators (ICDs) are routinely used in clinical medicine, but electrical shocks can cause significant side effects including tissue damage and traumatic pain. Recently, a new strategy for arrhythmia control using low-energy antifibrillation pacing (LEAP) was developed. LEAP uses a series of five low-energy pulses to terminate atrial fibrillation *in vivo* in Beagle dogs, permitting an average energy reduction of 84% compared to a single standard defibrillation shock, as well as 85% energy reduction for VF *in vitro* (Luther et al., 2011). LEAP exploits endogenous electrophysiological and structural properties of the heart: low-energy electric field pulses induce emission of waves from heterogeneities in electrical conductance, i.e., endocardial regions with high curvature and vascular tissue heterogeneities act as natural excitatory sources (“virtual electrodes”). These endogenous tissue sources target electrical turbulences near the cores of arrhythmia vortices, leading to successive synchronization and termination of fibrillation. To address 3D tissue properties, recordings of AP surface activity from panoramic imaging can provide valuable insights, when the reconstructed heart geometry is further aligned with computed tomography (CT) data. The combined strategy allows the identification of critical heterogeneities for virtual electrode recruitment while performing experiments with varying stimulation electrode geometries and field strengths. Moreover, *in vivo* experiments only permit an overall evaluation of the success rates of LEAP for ensemble parameter combinations (e.g., number, energy, and timing of pulses), while *ex vivo* imaging additionally facilitates detailed analysis about the time and location of residual chaotic activity in the heart. Finally, detailed experimental knowledge about the electrical activity on the cardiac surface together with internal tissue structures can be combined through 3D computational methods to reconstruct the hidden spatiotemporal dynamics of arrhythmias and therapeutic interventions inside the heart tissue in a non-invasive manner.

## Summary and conclusions

In summary, panoramic imaging of rabbit and larger mammalian hearts has been successfully developed whereas panoramic imaging of smaller, particularly transgenic mouse hearts will most likely be realized in the near future based on the success of VSDI and multiparametric readouts such as combined Ca^2+^ transients. Recent data from transgenic mouse hearts identified novel molecular determinants of ion channel expression and targeting in cardiac myocytes with marked effects on the conduction properties of the ventricles. This led to the realization that complex molecular mechanisms underlie the anisotropic activation pattern of the intact hearts, namely ion channel complexes at gap junctions, lateral membranes, and T-tubules, and all with specific effects on conduction properties. In general, acquired or genetic changes result in decreased spatiotemporal AP propagation and conduction delay, which may contribute to arrhythmogenesis. Panoramic imaging of mouse and larger hearts will contribute to better understanding of arrhythmia propensity and onset, as well as support clinical translation of important new therapeutic concepts, in particular 3D-MIMS and LEAP.

### Conflict of interest statement

The authors declare that the research was conducted in the absence of any commercial or financial relationships that could be construed as a potential conflict of interest.
